# Benign Episodic Mydriasis as a Cause of Isolated Anisocoria

**DOI:** 10.5811/cpcem.1316

**Published:** 2023-04-18

**Authors:** Alyssa Seibold, Jonathan Barnett, Lawrence Stack, Charles Lei

**Affiliations:** * Vanderbilt University Medical Center, Department of Emergency Medicine, Nashville, Tennessee; † Vanderbilt University Medical Center, Department of Ophthalmology and Visual Sciences, Nashville, Tennessee

**Keywords:** benign episodic mydriasis, anisocoria, migraine headache

## Abstract

**Case Presentation:**

A 22-year-old female presented to the emergency department with a dilated right pupil and mild blurry vision. Physical examination revealed a dilated, sluggishly reactive right pupil, without other ophthalmic or neurologic abnormalities. Neuroimaging was normal. The patient was diagnosed with unilateral benign episodic mydriasis (BEM).

**Discussion:**

BEM is a rare cause of acute anisocoria with an underlying pathophysiology that is not well understood. The condition has a female predominance and is associated with a personal or family history of migraine headaches. It is a harmless entity that resolves without intervention and results in no known permanent damage to the eye or visual system. Benign episodic mydriasis is a diagnosis of exclusion that may be considered only after the life- and eyesight-threatening causes of anisocoria.

## CASE PRESENTATION

A 22-year-old female with no known medical history presented to the emergency department (ED) with a dilated right pupil and mild blurry vision that she first noticed after waking up in the morning. She reported no eye pain or redness, double vision, or headache. She had no history of trauma to the eye, prior ocular disease, or known exposure to medications or toxins. Her vital signs were normal, including a blood pressure of 115/59 millimeters (mm) of mercury. Her visual acuity was 20/20 in both eyes. Her right pupil was round, 7 mm in diameter (Image), and sluggishly reactive to light, while her left pupil was 4 mm and briskly reactive. She had no afferent pupillary defect. She had intact extraocular movements, full visual fields, and normal intraocular pressures in both eyes.

She had no ptosis or other neurologic deficits. Computed tomography angiography of the head and neck was normal. She was evaluated in the ED by an ophthalmologist who felt her presentation was most consistent with unilateral benign episodic mydriasis (BEM). The mydriasis resolved prior to her follow-up ophthalmology appointment three weeks later, and she had no recurrences.

## DISCUSSION

The causes of anisocoria (greater than 1 mm difference in pupillary diameter) range from benign conditions to life- or eyesight-threatening emergencies. In patients presenting to the ED with acute anisocoria, it is imperative to first consider the most serious etiologies, including cerebral aneurysm, stroke, intracranial hemorrhage, meningitis, intracranial or ocular mass, acute angle-closure glaucoma, open globe, optic neuritis, and ocular infections. Benign causes of acute anisocoria include BEM, traumatic mydriasis, post-surgical changes, ocular migraine, and medication or chemical exposure (eg, anticholinergics, sympathomimetics).

The first step in the evaluation of isolated anisocoria is to determine which pupil is abnormal.^1^ If the larger pupil is found to be abnormal and a third nerve palsy has been excluded, pilocarpine may be used to determine the cause of unilateral mydriasis. Pupillary constriction in response to dilute (0.1%) pilocarpine indicates Adie tonic pupil. In the absence of a response to dilute pilocarpine, concentrated (1%) pilocarpine may be administered; pupillary constriction indicates BEM, whereas the lack of a response suggests pharmacologic mydriasis.

Benign episodic mydriasis is a rare cause of acute anisocoria and a diagnosis of exclusion. While typically unilateral, the affected eye may alternate in recurrent episodes and there may be bilateral involvement during a single episode.^2^ Patients with BEM may have isolated anisocoria or experience a wide variety of concomitant symptoms, such as blurry vision, photophobia, orbital pain, nausea, eye redness, double vision, or headache.^3^

The underlying pathophysiology of BEM is not well understood. Several studies have suggested that BEM may be caused by hyperactivity of the sympathetic nervous system (which causes pupillary dilation) or hypoactivity of the parasympathetic nervous system (which causes pupillary constriction).^4^ It occurs predominantly in females and has been connected to a personal or family history of migraine headaches, particularly in patients with recurrent episodes, but the significance of these associations is not clear.^2^,^3^

An episode of BEM may resolve within minutes to hours or persist for weeks to months.^3^ Aside from the cosmetic inconvenience and discomfort of migraine-associated symptoms (if present), BEM is a harmless entity, and there is no known irreversible damage to the eye or visual system.^2^

*CPC-EM Capsule*

What do we already know about this clinical entity?

*The causes of anisocoria range in seriousness from benign conditions to life- or eyesight-threatening emergencies.*

What is the major impact of the image(s)?

*The image illustrates a case of benign episodic mydriasis, a rare cause of acute anisocoria that has not been well described in the emergency medicine literature.*

How might this improve emergency medicine practice?

*Emergency physicians should have a stepwise approach to the evaluation of isolated anisocoria and consider benign episodic mydriasis as a diagnosis of exclusion.*

## Figures and Tables

**Image:**
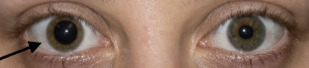
The patient’s right pupil (arrow) was dilated and sluggishly reactive to light, compared to her normal left pupil.
